# Far Infrared Radiation Attenuates Bleomycin-Induced Pulmonary Fibrosis in Mice via Modulation of the p53/TGF-β Signaling Pathway

**DOI:** 10.3390/ijms27062551

**Published:** 2026-03-10

**Authors:** Jicheng Li, Jingxu Chang, Wenhan Chu, Yu Jiang, Shaodi Sun, Xiaodi Ding, Liying Zhang, Lihong Shi

**Affiliations:** 1 School of Clinical Medicine, Shandong Second Medical University, Weifang 261053, China; 19506585856@163.com (J.L.); 15505335837@163.com (J.C.);; 2 School of Rehabilitation Medicine, Shandong Second Medical University, No. 7166, Baotong West Street, Weifang 261041, China; 3 Department of Clinical Pathology, School of Basic Medicine, Shandong Second Medical University, Weifang 261053, China

**Keywords:** far infrared radiation, pulmonary fibrosis, inflammation, fibroblast-to-myofibroblast differentiation, epithelial–mesenchymal transition, angiogenesis, p53, TGF-β1

## Abstract

Currently, there is no curative medication for idiopathic pulmonary fibrosis (IPF), and therapeutic interventions for IPF are hindered by limited efficacy and severe adverse side effects. Far Infrared Radiation (FIR), an invisible form of electromagnetic energy, has garnered increasing attention for its multiple biological effects. However, its therapeutic benefits and the underlying mechanisms of IPF have not been investigated. In the present study, we established a mouse model of bleomycin-induced pulmonary fibrosis (BIPF) to assess the efficacy of FIR in attenuating BIPF. The results showed that FIR therapy significantly improved the general condition of the mice and protected pulmonary function by ameliorating lung fibrosis, collagen deposition and excessive inflammation. Moreover, FIR could alleviate fibroblast-to-myofibroblast differentiation (FMD), the epithelial–mesenchymal transition (EMT) and angiogenesis in BIPF mice. These beneficial effects were notable both in the pro-fibrotic inflammatory stage and the following fibrotic stage. Mechanistically, FIR exerted anti-inflammatory and anti-fibrotic effects through modulating the p53/TGF-β signaling pathway. Overall, this study elucidates the anti-fibrotic activity and the potential molecular mechanisms of FIR in treating BIPF, providing a therapeutic strategy of convenient, non-invasive physical therapy for alleviating IPF. Of greater significance, the findings of this study display the promising future applications of FIR in managing the physiopathology of various chronic diseases.

## 1. Introduction

IPF is a progressive lethal disease characterized by chronic lung inflammation, pulmonary fibrosis, excessive accumulation of the extracellular matrix (ECM) and impaired lung function [1,2]. Despite extensive research efforts, effective therapies remain scarce. Although nintedanib and pirfenidone have been approved by the United States Food and Drug Administration (FDA) for IPF treatment [3], owing to the complex pathological mechanisms of IPF, the effectiveness of both nintedanib and pirfenidone is limited, and both are accompanied by major adverse reactions [4]. Therefore, it is imperative to develop effective treatments for IPF [5].

Although organ fibroses are often non-resolving in humans and the precise initiating factors for human IPF are not well clarified, a large number of studies have shown that therapies can be identified by studying mechanisms of therapeutic measures in mice [6]. To our knowledge, BIPF is the best mouse model for studying IPF [7]. BIPF mice exhibit severe fibrotic manifestations and fatal damage to respiratory function, which generalizes to the progressive fibrotic pathophysiology in the lungs despite the imperfect recapitulation of IPF [8].

FIR, an invisible form of electromagnetic solar energy with a wavelength ranging between 3.0 and 1000 μm, has garnered increasing attention for its potential therapeutic effects [9]. According to thermodynamics and quantum mechanics, all objects above absolute zero (−273.15 °C) can both emit and receive electromagnetic waves. It is interesting that the wavelength emitted by the human body is exactly part of FIR [10]; thus, the biological effects of FIR have aroused great attention [11]. As a convenient, non-invasive, non-contact physical therapy, FIR can be absorbed into deep tissues to relieve pain, promote healing, modulate inflammation and improve circulation [12,13]. FIR has been shown to exert protective effects against multiple inflammatory and fibrotic diseases by modulating TGF-β/p53/NF-κB-mediated inflammation and fibrogenesis. In murine models of acute respiratory distress syndrome (ARDS), FIR attenuated severe pulmonary inflammation and improved survival by inhibiting IL-1β, IL-6 and NF-κB/p65 signaling [14,15]. In addition, FIR could ameliorate hepatic fibrosis in metabolic dysfunction-associated fatty liver disease via suppressing TGF-β signaling, as well as alleviate cisplatin-induced vascular damage and nephrotoxicity by regulating NF-κB [16,17]. Therefore, we wondered if FIR could attenuate bleomycin-induced pulmonary fibrosis by modulating p53/TGF-β-mediated inflammatory and fibrotic pathological response.

In the present study, we exploited a mouse model of BIPF to identify the effect and mechanism of FIR in mitigating pulmonary fibrosis. We certified that FIR could suppress lung fibrosis by inhibiting FMD, EMT, angiogenesis and excessive inflammation to retard the progressive lesions of BIPF. These findings provide insight into the prospective application of FIR in treating IPF.

## 2. Results

### 2.1. FIR Protects Against BLM-Induced Fibrotic Lung Damage in Mice

**Figure 1:**
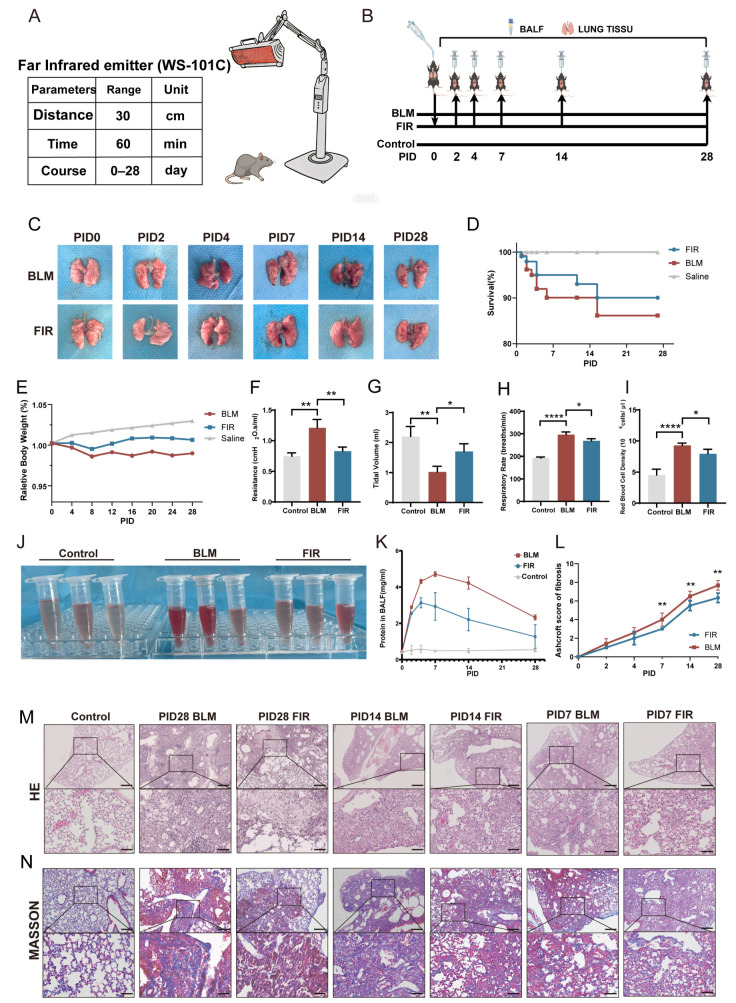
FIR treatment ameliorated BIPF in mice and improved pulmonary function. (**A**) FIR emitter device WS-101C. (**B**) Flowchart of this study to investigate the effect of FIR on BLM-induced PF mouse model (5 mg/kg). (**C**) Lungs of BLM and FIR mice on PIDs 0, 2, 4, 7, 14, 28 respectively. (**D**) Survival rate of BLM and FIR mice at representative time points. (**E**) Relative body weight (vs. baseline body weight) at the indicated time. (**F**–**H**) Airway resistances, tidal volume and respiratory rate of control, BLM and FIR mice on PID 28. (**I**) Red blood cell count in BALF from control, BLM and FIR mice on PID 28; (**J**) Macroscopic observation of the content of red blood cells in BALF from control, BLM and FIR mice respectively on PID 28. (**K**) Concentrations of total protein in BALF measured by BCA Protein Assay kit. (**L**) Ashcroft score of BLM and FIR mice estimated by HE and Masson images of lung slices. (**M**,**N**) Representative HE staining images of lung slices of BLM and FIR mice (upper scale bar, 200 μm, and lower scale bar, 50 μm) and Masson staining (each image’s upper scale bar: 400 μm; lower scale bar: 25 μm) on PIDs 7, 14, 28. Results are shown as mean ± SEM (*n* = 6). * *p* < 0.05, ** *p* < 0.01, **** *p* < 0.0001.

In this work, the effects of FIR treatment on the progression of BIPF in mice were explored. Therapeutic parameters of WS-101C are shown in Figure 1A, and the animal experimental model and procedure are shown in the flowchart of Figure 1B. The typical condition of the mice was observed and recorded after BLM was intratracheally administered. Six mice in each group were sacrificed for lung tissue samples on post-injury days (PIDs) 0, 2, 4, 7, 14, and 28, respectively. Gross observation of the lung samples at different time points revealed more severe, extensive fibrosis in the lungs of BLM mice than those of FIR mice (Figure 1C). During PID 0–7, mice in both the BLM and FIR groups had dull fur, less activity, slow response, shortness of breath accompanied by coughing, poor appetite, and decreased body weight. Most of the deceased mice died within the first week, and the mortality of FIR mice was lower than that of BLM mice, while there were no deaths in the control mice (Figure 1D). From PID 8, the typical condition of FIR mice improved gradually, and there was an obvious difference in body mass between the FIR and BLM mice on PID 28 (Figure 1E).

More importantly, on PID 28, pulmonary function testing showed that the tidal volume of FIR mice was much better than that of BLM mice, while the respiratory rate and lung resistance of FIR mice were obviously lower than those of BLM mice, indicating better lung tissue flexibility and lung function of FIR mice than in BLM mice (Figure 1F–H). In addition, erythrocyte counting performed on BALF collected on PID 28 showed lower red blood cell numbers in FIR mice than in BLM mice, suggesting less congestion in the lung tissues in the FIR group (Figure 1I,J). Moreover, plasma leakage into BALF as a proxy for lung damage was assessed by BCA assay, and FIR-treated mice displayed significantly lower albumin levels compared to BLM mice (Figure 1K).

One of the pathological features of lung fibrosis is excessive aggregation of fibroblasts, which leads to a reduction in lung compliance and impairment of gas exchange [18]. This pathological change immediately began to develop after BLM administration and was most pronounced on PID 28. Hematoxylin and eosin (HE) and Masson staining were used to observe the histological architecture of lung slices. It was obvious that the overall fibrotic area of lung tissues in FIR mice was decreased compared to BLM mice (Figure 1M). Moreover, Masson staining showed a noticeable reduction of collagen deposition in the lung tissues of FIR mice compared to those of BLM mice (Figure 1N). Consistently, Ashcroft scoring also showed that the FIR treatment obviously alleviated inflammatory infiltration and collagen deposition on PIDs 7, 14, and 28, indicating the promising effect of FIR in mitigating BIPF (Figure 1L).

### 2.2. FIR Alleviates FMD, EMT and Angiogenesis in BLM-Induced Lung Fibrogenesis

**Figure 2:**
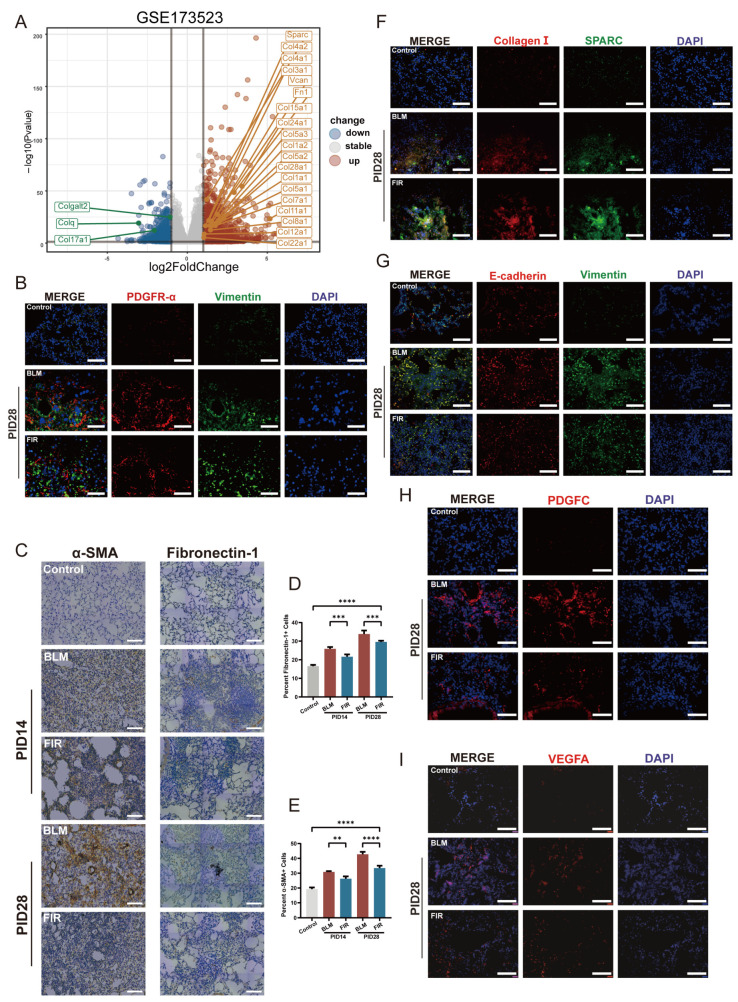
FIR treatment effectively alleviated FMD, EMT and angiogenesis in BLM-induced lung fibrogenesis. (**A**) Gene expression levels were analyzed by bulk RNA-seq in IPF mice. ECM-related genes were highlighted using public transcriptomic data from the GEO database (GSE173523). (**B**) Representative immunofluorescence staining of PDGFR-α (red) and Vimentin (green) in lung slices of Control, BLM and FIR mice on PID 28 (scale bar: 75 μm). (**C**) Representative immunohistochemical staining of α-SMA and Fibronectin-1 in lung slices of control, BLM and FIR mice respectively on PIDs 14 and 28 (scale bar: 75 μm). (**D**,**E**) The quantification of Collagen I, α-SMA, Fibronectin-1 positive cells respectively in lung slices. Results are shown as mean ± SEM (*n* = 6). ** *p* < 0.01, *** *p* < 0.001, **** *p* < 0.0001. (**F**) Representative immunofluorescence staining of Collagen I (red) and SPARC (green) in lung slices of control, BLM and FIR mice respectively on PID 28 (scale bar: 75 μm). (**G**) Representative images of immunofluorescence staining of E-cadherin (red) and Vimentin (green) in lung slices of control, BLM, and FIR mice respectively on PID 28 (scale bar: 75 μm). (**H**,**I**) Representative immunofluorescence staining of PDGFC (red) and VEGFA (red) in lung slices of control, BLM and FIR mice on PID 28 (scale bar: 75 μm).

During fibrogenesis of BIPF, injury-activated fibroblasts and FMD play crucial roles by replacing resident cells, disrupting lung structure and causing excessive deposition of ECM (Figure 2A) [19]. Under bleomycin injury, fibroblasts typically undergo phenotypic differentiation into two distinct subpopulations: activated fibroblasts and myofibroblasts. These two phenotypic differentiations usually become most pronounced on PID 28, characterized by robust expression of the typical biomarkers platelet-derived growth factor receptor-α (PDGFR-α) and Vimentin in activated fibroblasts, and α-smooth muscle actin (α-SMA) and Fibronectin-1 in myofibroblasts [20,21]. Immunofluorescence (IF) and immunohistochemistry (IHC) assays showed that the expressions of PDGFR-α, Vimentin, α-SMA, Fibronectin-1 (Figure 2B,C) and the ECM components Collagen I and Secreted Protein Acidic Rich In Cysteine (SPARC) (Figure 2F) were significantly upregulated in BLM mice. Interestingly, FIR effectively downregulated the expression of the abovementioned fibroblast differentiation-related markers and ECM components, indicating that FIR could inhibit fibroblast activation, FMD, and ECM deposition in fibrotic lesions.

It is noteworthy that, in addition to FMD, alveolar epithelial cells (AECs) are also capable of transdifferentiating into pathological fibroblasts via EMT [22]. To explore if that FIR could prevent the EMT of AECs, we analyzed the expression of E-cadherin and Vimentin by double immunofluorescence. The results showed that FIR treatment significantly increased E-cadherin level and decreased Vimentin level in BLM-induced fibrotic lung tissues, suggesting that FIR could inhibit the EMT during the development of BIPF (Figure 2G).

It cannot be ignored that the development of pulmonary fibrosis is accompanied by a paradoxical phenomenon in fibrotic lesions: the loss of existing microvasculature occurs concomitantly with the initiation of aberrant angiogenesis [23]. Appropriate angiogenesis helps to improve the microcirculation within the fibrotic foci whereas excessive angiogenesis can facilitate lung fibrosis. Both platelet-derived growth factor-C (PDGFC) and vascular endothelial growth factor A (VEGFA) are indispensable indicators of newly formed capillaries [24,25]. To observe if FIR treatment could modulate angiogenesis, we detected the expressions of PDGFC and VEGFA in lung tissues. Intriguingly, we found that FIR treatment could prominently inhibit the over-expression of PDGFC and VEGFA caused by BLM (Figure 2H,I). Overall, all these results supported that FIR treatment could effectively suppress the major pathogenic FMD, EMT and angiogenesis in lung fibrogenesis.

### 2.3. FIR Restrains Excessive Inflammation in Lung Tissues Induced by Bleomycin

**Figure 3:**
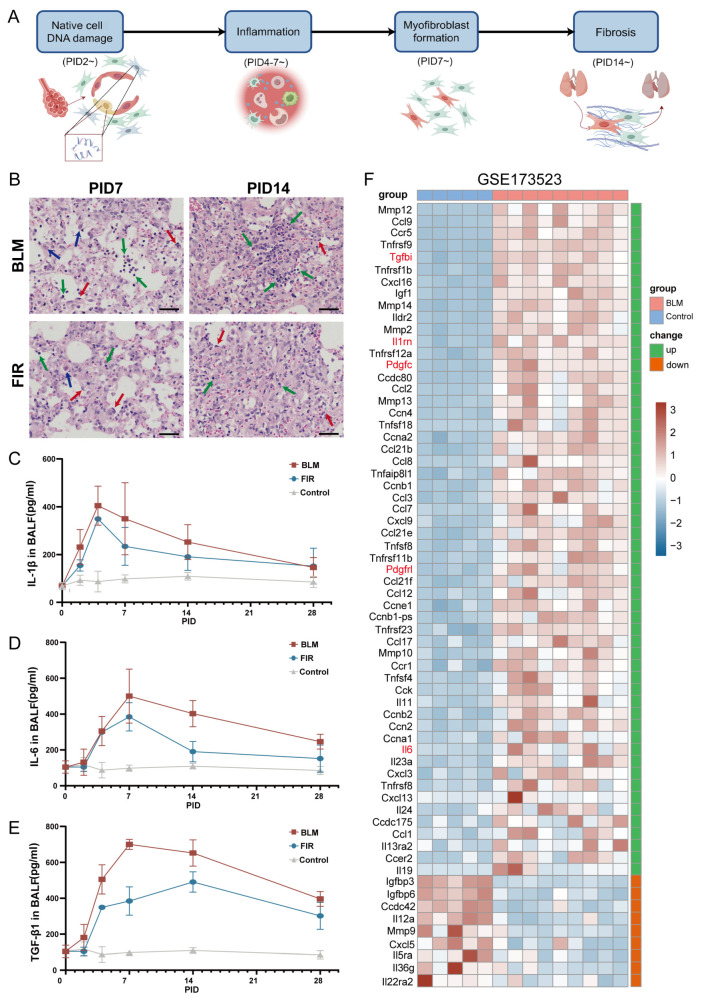
FIR treatment suppressed the excess inflammation in BIPF. (**A**) Flowchart of the pathophysiologic characteristics of BIPF at different timepoints in mice. (**B**) H&E staining of representative lung slices from BLM and FIR mice on PID 7 and PID 14. The infiltration of inflammatory cells was indicated with red arrows (alveolar epithelial cells), blue arrows (macrophage), green arrows (neutrophil) (scale bar: 50 μm). (**C**–**E**) ELISA assay for IL-1β, IL-6, TGF-β1 in BALF obtained on PIDs 0, 2, 4, 7, 14, 28 (*n* = 6). (**F**) Heatmap visualization of senescence-associated secretory phenotype (SASP)-associated genes via bulk RNA-seq of control and BLM (PID 21) mice from public transcriptomic data from the GEO database (GSE173523) (Benjamini–Hochberg method).

BLM can cause damage to native cells in the lungs, including AECs, fibroblasts and basal cells. The damaged cells thus activate and recruit immune cells, which in turn secrete a repertoire of pro-fibrotic inflammatory cytokines, an effect that is particularly pronounced on PIDs 4–7 [26,27]. This cascade of events ultimately drives the excessive proliferation and accumulation of fibroblasts, as well as aberrant ECM deposition, a pathological process that is characteristically evident from PID 14 onwards [28] (Figure 3A). Macroscopic observation and the red blood cell (RBC) count in BALF from the BLM group further corroborated the presence of markedly enhanced plasma extravasation and prominent RBC infiltration into the alveolar spaces of the lung parenchyma of mice with BIPF (Figure 1I,J). In order to observe whether FIR could restrain the excessive inflammation induced by bleomycin, H&E staining was used to detect the infiltration of inflammatory cells in lung tissues on PID 7 and PID 14. The results showed that there was less infiltration of inflammatory cells in the lung tissues of FIR mice than those of BLM mice (Figure 3B). To further explore the influence of FIR treatment on the abnormal inflammatory response following bleomycin instillation, BALF (PIDs 0, 2, 4, 7, 14, and 28) was collected, and the levels of inflammatory cytokines were detected by ELISA. The results showed that interleukin-1β (IL-1β) and interleukin-6 (IL-6), which are primary inflammatory cytokines contributing to fibrosis, as well as transforming growth factor-β1 (TGF-β1), a typical pro-fibrotic factor, were significantly upregulated in BLM-treated mice (Figure 3C–F). It is exciting to find that FIR could effectively restrain the upregulation of IL-1β, IL-6 and TGF-β1 at different PID points. Notably, the concentrations of TGF-β1 in FIR mice remained at relatively higher levels until PID 28 than those of the control group. Moreover, FIR delayed the peak time of TGF-β1 from PID 7 to PID 14, whereas the peak times of IL-1β (PID 2) and IL-6 (PID 7) were not changed (Figure 3C,D). Accordingly, these results suggest protective benefits of FIR in modulating excessive inflammation caused by BLM.

Recent studies showed that various cell types, including fibroblasts, epithelial cells and macrophages, exhibit a morphological senescence-like phenotype in fibrotic lung tissues [29]. The pathological features of BIPF, such as DNA damage and continuous inflammatory response, are also characteristics of cell senescence [30,31]. These senescent cells typically exhibit a senescence-associated secretory phenotype (SASP), characterized by the secretion of inflammatory factors including IL-1β, IL-6 and TGF-β1, which are consistent with our findings in BALF [32,33]. Consequently, we explored the bulk RNA-sequencing GEO database and found a close correlation between BIPF and SASP-associated-genes (Tgfbi, Il1rn, Pdgfc, Il6) in mice (Figure 3F), further supporting that FIR could repress excessive inflammatory responses in BIPF.

### 2.4. FIR Alleviates BIPF Through Mediating p53/TGF-β Pathway

**Figure 4:**
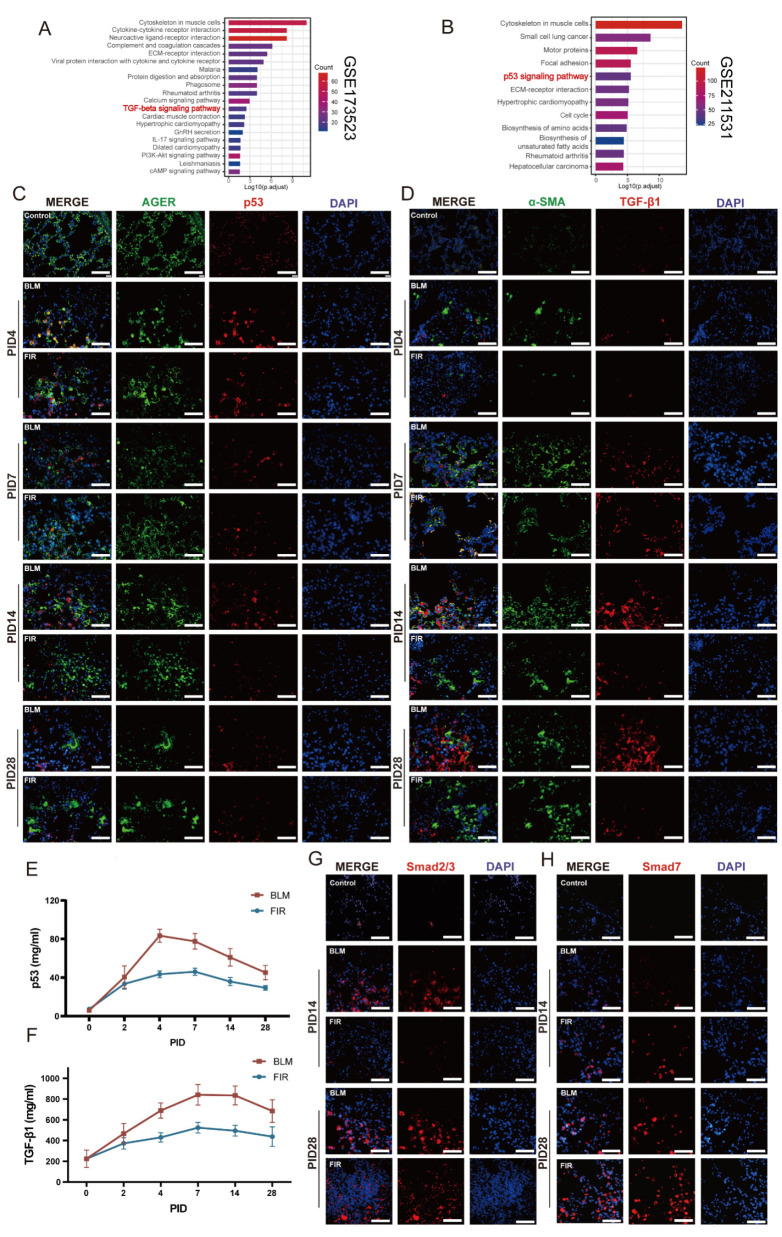
FIR treatment attenuates BIPF in mice via modulation of p53/TGF-β signaling pathway. (**A**) KEGG pathway enrichment analysis of DEGs in BIPF mice on PID 21 using public transcriptomic data from the GEO database GSE173523. (**B**) KEGG pathway enrichment analysis of DEGs in mice alveolar organoids 48 h after bleomycin treatment using public transcriptomic data from the GEO database GSE211531. (**C**) Representative immunofluorescence staining of p53 (red) and AGER (green) in lung slices of control, BLM, and FIR mice on PIDs 4, 7, 14, 28 (scale bar: 75 μm). (**D**) Representative immunofluorescence staining of TGF-β1 (red) and α-SMA (green) in lung slices of control, BLM and FIR mice on PIDs 4, 7, 14, 28. (**E**,**F**) ELISA assay for p53, TGF-β1 contents in mice lung tissue obtained on PIDs 0, 4, 7, 14, 28 (*n* = 6). (**G**,**H**) Representative immunofluorescence staining of Smad2/3 (red) and Smad7 (red) in lung tissues of control, BLM and FIR mice on PID 28 (scale bar: 75 μm).

In order to explore the potential mechanisms underlying the therapeutic effect of FIR on BIPF, we performed bioinformatic analysis using public transcriptomic data from the GEO database (GSE173523). KEGG pathway enrichment analysis showed that the calcium, TGF-β, IL-17, PI3K-Akt, and cAMP signaling pathways were highly enriched in BIPF mice on PID 21 (Figure 4A), among which TGF-β/Smad signaling is a crucial pathway in mediating the pathogenic process of BIPF [34,35]. Another interesting and remarkable finding is that p53, one of the upstream transcription factors of TGF-β, is enriched at the beginning of BIPF (mouse alveolar organoids cultured for 48 h from public transcriptomic data of the GEO database GSE211531) (Figure 4B) and there is a time lag between p53 activation (PID4) and the upregulation of TGF-β-related profibrotic genes (PID 7) [36,37]. Then we examined the expression of p53 and TGF-β signaling in the lung tissues of BLM-treated mice on PIDs 4, 7, 14, and 28 respectively. Consistent with the results of the bioinformatic analysis and previously reported studies [38,39], we found a higher expression of p53 in PID 4 lung tissues (Figure 4C). It is noteworthy that high expression of advanced Glycosylation End-Product Specific Receptor (AGER), which is a marker of Alveolar type I (ATI) cells, simultaneously coexisted with high expression of p53, further indicating that p53 is enriched at the early pro-fibrotic stage after BLM injury, with alveoli with relatively normal structures [40]. In contrast, in lung tissues on PIDs 7, 14, and 28, there were relatively lower expressions of p53 and higher expressions of TGF-β1 (Figure 4E,F), consistent with our finding that TGF-β1 had a delayed peak time in BALF. More importantly, these results also supported the theory that there was a time lag between the activation of p53 and the consequent activation of the TGF-β/Smad pathway. Moreover, it was of great significance that the FIR mice had lower expression levels of p53, TGF-β1 and Smad2/3 than the BLM mice at different time points (Figure 4G), suggesting that FIR could modulate the function of the TGF-β/Smad pathway. Meanwhile, the finding that Smad7 in FIR mice was remarkably higher than in BLM mice further provided evidence for the therapeutic effect of FIR in mitigating IPF (Figure 4H).

## 3. Discussion

Continuous chronic damage, delayed aberrant healing and persistent intensive inflammatory response are key features of IPF. Despite the progress in understanding the pathogenesis of IPF and the development of IPF therapies [41,42], currently, there are no curative medications, and therapeutic approaches cannot retard the progression of the disease [43].

FIR possesses a vibrational energy level similar to that of biological macromolecules, generating comprehensive and systematic resonant effects in organisms [44]. As a multi-target physical therapy, FIR irradiation affects the molecular bonds of metabolic substances, regulates extracellular matrix components (e.g., collagen and keratin) and intracellular substances (e.g., glycogen), alters water’s physicochemical properties (e.g., smaller water clusters and higher dielectric constant), and mediates the activation or inhibition of TGF-β, NF-κB, AMPK, and oxidative stress pathways [14,45,46]. Studies have shown that FIR generates a variety of biological effects through non-thermal effects, promoting tissue regeneration, alleviating fibromuscular pain, accelerating skeletal muscle function recovery, and regulating inflammatory responses [47,48]. However, its effect on pulmonary fibrosis remains uninvestigated. The present study demonstrated that FIR treatment inhibits BIPF.

First of all, FIR attenuated abnormal inflammatory responses by inhibiting the secretion of IL-1β, IL-6 and TGF-β1. IL-1β and IL-6 are primarily produced by M1 macrophages, which drive the inflammatory cascades in IPF [49]. TGF-β1 is one of the most potent profibrotic mediators, released from macrophages, infiltrating regulatory T cells and alveolar epithelial cells after injury. The overproduction of TGF-β1 leads to tissue fibrosis by recruiting inflammatory cells and increasing the differentiation of fibroblasts, as well as the deposition of ECM [50,51]. TGF-β signaling transduces via Smad-dependent canonical pathway to regulate cell proliferation, differentiation, immune response and angiogenesis [52,53]. Remarkably, our research confirmed the pivotal role of the TGF-β pathway in a BIPF mouse model and verified the upregulation of TGF-β1 and Smad2/3 and downregulation of Smad7 in response to BLM exposure.

Secondly, consistent with a previous report that p53 is positively correlated with TGF-β1, collagen I, and α-SMA in human lung tissues [54], our finding confirm the enrichment of p53 at the beginning of BIPF (PID4) and the time lag between p53 activation and TGF-β overexpression (PID 7) [36,37]. TGF-β1 and p53 perform extensive, bidirectional regulation of cell fate, proliferation/apoptosis, EMT, and tissue remodeling. In physiological conditions, p53 and TGF-β1 are mostly “antagonistic” in maintaining tissue homeostasis, whereas under pathological microenvironmental stimulation such as aging, inflammation, and tumors, p53 and TGF-β1 cooperate in driving tissue remodeling [19]. It is of great significance that FIR significantly inhibited the activation of the p53/TGF-β signaling pathway both in the early inflammatory stage and the following fibrotic stage.

Moreover, FIR could alleviate FMD, EMT and angiogenesis in BIPF. Bleomycin-induced injury led to fibroblast activation, fibroblast-to-myofibroblast differentiation and the EMT of AECs, resulting in lung structure disruption and ECM deposition. It was inspiring to find that FIR could significantly inhibit fibroblast activation, FMD, EMT, and ECM deposition in fibrotic lesions. In addition, disordered and immature pathological angiogenesis existing in fibrotic foci could facilitate the deterioration of the fibrotic lung structure [55,56], and our results verified that FIR can prominently inhibit excessive angiogenesis. In the BIPF model, bleomycin-induced injury and inflammation initiate pathological fibrosis and angiogenesis. Fibroblast transdifferentiation and abnormal angiogenesis exhibit a synergistic effect on pulmonary fibrosis, and it is also the core target of current anti-pulmonary fibrosis therapy.

Overall, as a convenient, non-invasive, non-contact physical therapy, FIR treatment significantly attenuated BIPF in murine models via modulation of the p53/TGF-β signaling pathway. Given the valuable findings of this study and the complex pathogenesis of pulmonary fibrosis, to improve the development of FIR-based therapies, further in-depth mechanistic investigations of its core molecular targets in both murine and translational human studies are warranted.

## 4. Materials and Methods

### 4.1. Animals

All procedures of the animal experiments conducted in this study were approved by the Institutional Animal Care and Use Committee of Shandong Second Medical University (2024SDL769). A total of 100 male C57BL/6 mice (5 weeks old) were purchased and housed in a standard pathogen-free (SPF) environment with a normal diet. To minimize procedural stress, all mice were acclimated to sham-FIR exposure (mice were placed under a non-emitting device) for 6 days before the start of the experiment. All mice were anaesthetized prior to single intratracheal administration of bleomycin (BLM) sulfate (Meilunbio, Dalian, China) at a dose of 2 mg/kg body weight (*n* = 94). A control group of 6 mice received an equal volume of sterile saline via intratracheal administration. A total of 94 BLM-treated mice were randomly divided into the BLM group and the FIR group, with FIR mice receiving FIR irradiation for 60 min daily for 28 days, from the first day of BLM instillation to PID 28, whereas BLM mice did not receive this treatment. According to the requirements of the American Veterinary Association’s Euthanasia Guidelines 2020, mice were euthanized on the indicated day via an excessive intraperitoneal injection of pentobarbital sodium, blood, and bronchoalveolar lavage fluid, and the lungs were quickly collected. A portion of the lung tissues were fixed with 4% paraformaldehyde, and the remaining lung tissues were stored at −80° for subsequent experiments.

### 4.2. FIR Treatment

An FIR emitter WS-101C (Zhoulin, Beijing, China) was kindly provided by Beijing Zhoulin Spectrum Technology Co., Ltd., which could generate electromagnetic waves with major wavelengths of 3.0 to 25 μm, which allows sufficient energy to reach deep organs while avoiding excessive surface heating or thermal damage [57]. The FIR irradiation instrument was turned on 5 min in advance to preheat, and irradiation treatment was started only when the temperature of the instrument stabilized at 40 ± 1 °C. The mice were placed at a distance of 30 cm from the FIR emitter and irradiated continuously for 60 min [46]. During the entire irradiation process, the skin surface temperature of the mice was continuously monitored to be stable at 40 ± 1 °C. The power density (irradiance) of FIR irradiation at the animal level was 42.5–54.2 mW/cm^2^.

### 4.3. Enzyme-Linked Immunosorbent Assay (ELISA)

Bronchoalveolar lavage fluid (BALF) was collected on PIDs 0, 2, 4, 7, 14, and 28, as described in previous reports [58]. The total protein concentrations of all collected BALF supernatants were measured by the BCA Protein Assay Kit (P0012S, Beyotime, Shanghai, China). The levels of TGF-β1, IL-6, and IL-1β in BALF supernatants were determined by a mouse enzyme-linked immunosorbent assay (ELISA) kit (KE10005, KE10007, KE10003, Proteintech, Wuhan, China) following the manufacturer’s protocols. Lung tissues on PIDs 0, 2, 4, 7, 14, and 28 were treated as described in previous reports [59]. BCA protein assays were conducted to determine total protein concentrations of corresponding samples. The levels of TGF-β1 and P53 in BALF supernatants were determined by the mouse ELISA kit (KE10005, Proteintech, Wuhan, China and ab224878, Abcam, Cambridge, UK, respectively) following the manufacturer’s protocols [60,61].

### 4.4. Histology Analysis

The fixed lung tissues embedded in paraffin were cut into 4 μm sections for HE staining, Masson staining, immunohistochemistry and immunofluorescent staining to evaluate the degree of fibrosis and collagen deposits. The results were scored by at least two professional pathologists. Quantification of lung sections was performed in at least 4 different fields in a random fashion manner.

### 4.5. Immunohistochemistry (IHC)

Immunohistochemistry was performed on paraffin-embedded lung tissue sections, as described in previous reports [62]. The primary antibodies used in this study were directed against Collagen I (1:400, AF7001, Affinity, Changzhou, China), α-SMA (1:400, AF1032, Affinity, Changzhou, China), Fibronectin-1 (1:400, BF9010, Affinity, Changzhou, China), and E-cadherin (1:5000, 20874-1-AP, Proteintech, Wuhan, China). Finally, the stained sections were observed and captured by optical microscopy (Olympus, Tokyo, Japan).

### 4.6. Immunofluorescent Staining (IF)

Immunofluorescent staining was performed on paraffin-embedded lung tissue sections, as described in previous reports [63]. The following primary aantibodieswere anti-E-cadherin (1:400, 20847-1-AP, Proteintech, Wuhan, China), anti-Fibronectin-1 (1:400, BF0273, Affinity, Changzhou, China), anti-Collagen I (1:400, AF7001, Affinity, Changzhou, China), anti-α-SMA (1:400, AF1032, Affinity, Changzhou, China), anti-TGF-β1 (1:400, 21898-1-AP, Proteintech, Wuhan, China), anti-Smad2/3 (1:400, AF6367, Affinity, Changzhou, China), anti-Smad7 (1:400, AF5147, Affinity, Changzhou, China), anti-SPARC (1:400, sc-73472, Santa Cruz, TX, USA), anti-PDGFC (1:400, 55076-1-AP, Proteintech, Wuhan, China), and anti-p53 (1:1000, BF8013, Affinity, Changzhou, China). Slides were washed with PBS and then incubated with secondary antibodies for 1 h at 37 °C. The secondary anti-bodies were anti-mouse IgG (1:1000, SA1000B-1, Proteintech, Wuhan, China) and anti-rabbit IgG (1:1000, SA1000B-4, Proteintech, Wuhan, China). The stained sections were scanned by confocal laser scanning microscope (TCS SP8, Leica, Germany).

### 4.7. Data Acquisition and Process

Two datasets (GSE173523, GSE211531) from the GEO database were downloaded and analyzed [7,36]. GSE173523 contained lung tissue bulk RNA sequencing data from nine PID 21-BLM and five saline-model mice. GSE211531 contained bulk RNA sequencing data of mouse alveolar organoids (48 h after bleomycin treatment vs. non-treatment). Differentially expressed genes (DEGs) were obtained by differential analysis of IPF and control samples, which were confirmed by the DESeq2 R package (v1.46.0). The cutoff values of adjusted *p*-value and |fold-change| were 0.05 and 2, respectively. The “ggplot2” (v3.5.1) and “pheatmap” (v1.0.12) packages were used to create the heatmap of TGF-β-associated DEGs. To understand the potential functional pathways and mechanisms of the disease, the “ClusterProfiler” R package (v4.10.0) was used for Kyoto Encyclopedia of Genes and Genomes (KEGG) pathway enrichment analysis as well as gene ontology (GO) enrichment analysis of DEGs. The top 10–15 enriched terms were visualized using a bar plot, and only terms with an adjusted *p*-value < 0.05 were considered statistically significant.

### 4.8. Statistical Analysis

Data are presented as the mean ± standard error (SE) of at least three independent experiments. Statistical analysis was performed using GraphPad Prism 9.5 software. An unpaired two-tailed Student’s *t*-test was used for comparisons between the two groups. For comparisons involving multiple groups and time points, two-way ANOVA followed by Tukey’s post hoc test was applied. A *p*-value < 0.05 was considered statistically significant.

## 5. Conclusions

This study revealed that FIR effectively improved pulmonary fibrosis by targeting and inhibiting multiple critical fibrogenic processes, including FMD, EMT, and angiogenesis, as well as the excessive inflammatory responses that are closely associated with lung epithelial cell dysfunction. Moreover, FIR irradiation markedly delayed the initiation and progression of the fibrotic process in BLM-induced IPF mice, exerting a notable protective effect on lung tissue integrity. Thus, these results clearly indicate that FIR holds considerable potential as a promising novel therapeutic mode for the clinical management of IPF by improving disease-related manifestations and delaying disease progression.

## Data Availability

The original contributions presented in this study are included in the article. Further inquiries can be directed to the corresponding author.

## Abbreviations

Idiopathic pulmonary fibrosis (IPF), extracellular matrix (ECM), bleomycin-induced pulmonary fibrosis (BIPF), Far Infrared Radiation (FIR), fibroblast-to-myofibroblast differentiation (FMD), epithelial–mesenchymal transition (EMT), post-injury day (PID), platelet-derived growth factor receptor-α (PDGFR-α), α-smooth muscle actin (α-SMA), Secreted Protein Acidic Rich In Cysteine (SPARC), alveolar epithelial cells (AECs), platelet-derived growth factor-C (PDGFC), vascular endothelial growth factor A (VEGFA), interleukin-1β (IL-1β), interleukin-6 (IL-6), transforming growth factor-β1 (TGF-β1), senescence-associated secretory phenotype (SASP).
